# Supplementary material for: Circulating Serum Cell-Free Mitochondrial DNA in Amyotrophic Lateral Sclerosis
Source: Cells. 2025 Sep 12;14(18):1433. doi: 10.3390/cells14181433 (PMC12468903; doi:10.3390/cells14181433)
Supplement: Supplementary file 1 [file cells-14-01433-s001.zip › cells-3795759-supplementary.pdf]

cells

MDPI

Supplementary Material

Circulating Serum Cell-Free Mitochondrial DNA in Amyotrophic Lateral Sclerosis

Giada Zanini [1], Ilaria Martinelli [2,*], Giorgia Sinigaglia [1], Elisabetta Zucchi [2,*], Federico Banchelli [2], Cecilia Simonini [2], Giulia Gianferrari [2,3], Andrea Ghezzi [2,3], Jessica Mandrioli [2,4] and Marcello Pinti [1]

[1] Department of Life Sciences, University of Modena and Reggio Emilia, 41125, Modena, Italy; giada.zanini@unimore.it (G.Z); giorgia.sinigaglia@unimore.it (G.S); marcello.pinti@unimore.it (M.P)
[2] Department of Neurosciences, Ospedale Civile di Baggiovara, Azienda Ospedaliero-Universitaria di Modena, 41126, Modena, Italy; federico.banchelli@gmail.com (F.B); ceciliasimonini24@gmail.com (C.S); giulia.gianferrari@unimore.it (G.G); andrea.ghezzi@unimore.it(A.G); jessica.mandrioli@unimore.it(J.M)
[3] Neuroscience PhD Program, Department of Biomedical, Metabolic and Neural Sciences, University of Modena and Reggio Emilia, 41125, Modena, Italy; giulia.gianferrari@unimore.it (G.G); andrea.ghezzi@unimore.it (A.G);
[4] Department of Biomedical, Metabolic and Neural Sciences, University of Modena and Reggio Emilia, 41125, Modena, Italy; jessica.mandrioli@unimore.it (J.M)
* Correspondance: martinelli.ilaria@aou.mo.it (I.M); zucchi.elisabetta@aou.mo.it (E.Z) Tel.:+390593961640 (E.Z)

Table S1. Association between cf-mtDNA, comorbidities and clinical characteristics in ALS patients of the study.

Variable	p-value
Age at onset	0.9181
Weight loss at sampling	0.4388
Site of onset	0.3333
Phenotype	0.6875
Depression	0.2635
Psychosis	0.2230
Parkinsonism	0.5965
COPD	0.8058
Other respiratory	0.9848
Diabetes	0.5571
Hypertension	0.4741
Cardiopathies	0.3766

Legend:: COPD: Chronic obstructive pulmonary disease.

Table S2. Biological indicators' distribution in ALS patients of the study.

Biomarker	N	Mean [SD]
NfL_{serum} pg/mL	52	116.88 [68.22]
$pNfH_{serum}$ pg/mL	53	1928.77 [1740.53]
$SerpinA1_{serum}$ ug/ml	51	1031.08 [561.27]
$TREM2_{serum}$ ng/mL	48	20.93 [10.89]
$CHIT3L1_{serum}$ ng/ml	52	60.91 [72.57]
NLR	53	2.13 [0.81]
MLR	53	0.27 [0.08]
SIRI	53	1.12 [0.51]
AISI	53	274.30 [145.87]

SII	53	252.61 [248.59]
Total Cholesterol mg/dL	50	207.52 [37.74]
HDL Cholesterol mg/dL	48	50.84 [14.93]
LDL Cholesterol mg/dL	48	137.89 [32.88]
Triglycerids mg/dL	49	110.37 [37.20]
Creatinine mg/dL	52	0.71 [0.20]

Notes: Means with standard deviations [SD] are reported between parentheses. **Legend**: NLR: Neutrophil-to-lymphocyte ratio, MLR: Monocyte-to-lymphocyte ratio, SIRI: systemic inflammation response index, AISI: Aggregate Systemic Inflammation Index, SII: systemic-immune- inflammation index.
